# Plant Proton Pumps and Cytosolic pH-Homeostasis

**DOI:** 10.3389/fpls.2021.672873

**Published:** 2021-06-09

**Authors:** Maike Cosse, Thorsten Seidel

**Affiliations:** Dynamic Cell Imaging, Department of Biochemistry and Physiology of Plants, Faculty of Biology, Bielefeld University, Bielefeld, Germany

**Keywords:** vacuolar-type ATPase, vacuolar pyrophosphatase, plasma membrane-ATPase, 14-3-3, redox

## Abstract

Proton pumps create a proton motif force and thus, energize secondary active transport at the plasma nmembrane and endomembranes of the secretory pathway. In the plant cell, the dominant proton pumps are the plasma membrane ATPase, the vacuolar pyrophosphatase (V-PPase), and the vacuolar-type ATPase (V-ATPase). All these pumps act on the cytosolic pH by pumping protons into the lumen of compartments or into the apoplast. To maintain the typical pH and thus, the functionality of the cytosol, the activity of the pumps needs to be coordinated and adjusted to the actual needs. The cellular toolbox for a coordinated regulation comprises 14-3-3 proteins, phosphorylation events, ion concentrations, and redox-conditions. This review combines the knowledge on regulation of the different proton pumps and highlights possible coordination mechanisms.

## Introduction

Central reactions, such as glycolysis, nitrate reduction, antero-, and retrograde signaling take place in the cytosol, but the cytosol is also a transit compartment for many solutes, which reside transiently in the cytosol subsequent uptake and before compartmentation into organelles. Though the pH in the cytosol is chemically buffered by bicarbonates, phosphate, and proteins, it is affected by other ions. pH-stat (7.1–7.5) is achieved physically by proton pumps and secondary active transport and chemically by metabolic processes, which either consume or release protons (Schumacher, 2014; Feng et al., 2020). The required pH in compartments and the apoplast might lead to a conflict with cytosolic pH-stat so that proton pumping alone is not sufficient. Then, cytosolic proton-scavenging by malate decarboxylation or glutamate decarboxylation allows for active chemical buffering and compensation for limited proton pumping. However, malate production releases four protons, so that *de novo* synthesis is counterproductive, while vacuolar malate storage ensures a backup of buffering capacity (Feng et al., 2020; Wegner and Shabala, 2020).

### The Plant Plasmamembrane Forms the Barrier to the Apoplast

The plant plasmamembrane forms the barrier to the apoplast. The apoplastic pH depends mainly on anion-channels, cation-antiporters, and plasma membrane-ATPase (PM-ATPase; Geilfus, 2017; Mangano et al., 2018). Cation channels like GORK1 co-operate with the PM-ATPase, too (van Kleeff et al., 2018), while opening of Kat1 depends on the apoplastic pH (Kazmierczak et al., 2013). Furthermore, clock-like oscillations were observed which might be connected to reactive oxygen species (ROS)-oscillations (Mangano et al., 2018). The pH-buffering capacity of the apoplast is only 10% of the cytosolic buffering capacity (Wegner and Shabala, 2020). This leads to rapid and transient fluctuations of the apoplastic pH and membrane potential. Apoplastic pH-alterations comprise both acidification and alkalization: for instance, apoplastic pH increases in leaves in response to stress factors, such as drought, salinity, and pathogens and might serve as systemic messenger. Alkalization probably involves proton uptake due to inhibition of the PM-ATPase or increased proton permeability. To terminate the signal, the apoplast becomes re-acidified within 2 h, reflecting re-activation of the PM-ATPase (Geilfus, 2017; van Kleeff et al., 2018). On the other hand, apoplastic acidification was observed in response to *Fusarium oxysporun*. Such pathogen-induced pH-changes regulate the growth defense and have a direct impact on pathogenicity (Kesten et al., 2019). Apoplast alkalinization is essential for growth of pollen tubes and root hairs (Mangano et al., 2018).

The vacuole is the main storage compartment for solutes and contributes up to 90% of the cellular volume, but the tonoplast contains just 1% of the cellular proteins, displaying the low abundance of transport protein in the cellular proteome (Fricke, 2017; Martinoia, 2018). Two types of proton pumps dominate at the tonoplast, comprising the vacuolar-type ATPase (V-ATPase) and the vacuolar pyrophosphatase (V-PPase), which energize transport at the tonoplast (Seidel et al., 2013). The acidic conditions in early endosomes (EE) and the trans-Golgi network (TGN) are required for proper transport and sorting. Altered pH homeostasis in TGN/EE disturbs cargo sorting and trafficking to vacuole by receptor-cargo interactions. Recently, Krebs and co-workers stated that the TGN/EE contributes to the uptake of solutes destined for the vacuole. In this scenario, the V-ATPase acidifies the lumen of the TGN and drives sodium-uptake in case of salinity (Krebs et al., 2010). NHX5 and 6 mediate K^+^/H^+^ exchange at the TGN/EE while CLC-d is capable to maintain the pH-gradient, regulating the luminal pH this way (von der Fecht-Bartenbach et al., 2007; Bassil et al., 2011). The vacuolar isoforms NHX1 and NHX2 serve as cation/proton antiporters (Sze and Chanroj, 2018), but are probably not essential for sodium sequestration (Barragán et al., 2012).

## Proton Pumps

The PM-ATPase utilizes ATP as energy source (Wegner and Shabala, 2020) and drives solute uptake and water uptake at the plasmamembrane (Fricke, 2017), thereby having an impact on phloem loading, metabolite transport, and growth and nutrient uptake and distribution (Briskin and Hanson, 1992; Morsomme and Boutry, 2000; Palmgren, 2001; Sondergaard et al., 2004; Zhao et al., 2008). Together with anion channels, the PM-ATPase functions in the re-acidification of the apoplast subsequent alkalization (Geilfus, 2017; Feng et al., 2020), while hyperpolarization is driven by PM-ATPase under acid stress, followed by electrical balancing by potassium symporters and channels (Sze and Chanroj, 2018). In guard cells, the PM-ATPase is of particular importance, since it is involved in stomatal closure with respect to environmental factors, for instance in pathogen-induced stomatal closure (Kaundal et al., 2017). Eleven isoforms (AHA1–11) of the PM-ATPase are known in *Arabidopsis thaliana*. Except of AHA10, which was found at the tonoplast in the endothelium, all members locate to the plasma membrane (Appelhagen et al., 2015; Li et al., 2016). The PMF generated by AHA1 and AHA2 is essential and a double knock-out of both turned out to be embryolethal (Mangano et al., 2018; Sze and Chanroj, 2018). In addition to blue-light-dependent activation, AHA2 requires light for proper transport to the plasma membrane and locates to endomembranes with dim light (Haruta et al., 2018). AHA7 senses the apoplastic pH in root epidermal cells *via* an extracellular loop and represses proton pumping as negative feedback regulation (Hoffmann et al., 2019).

**Figure 1:**
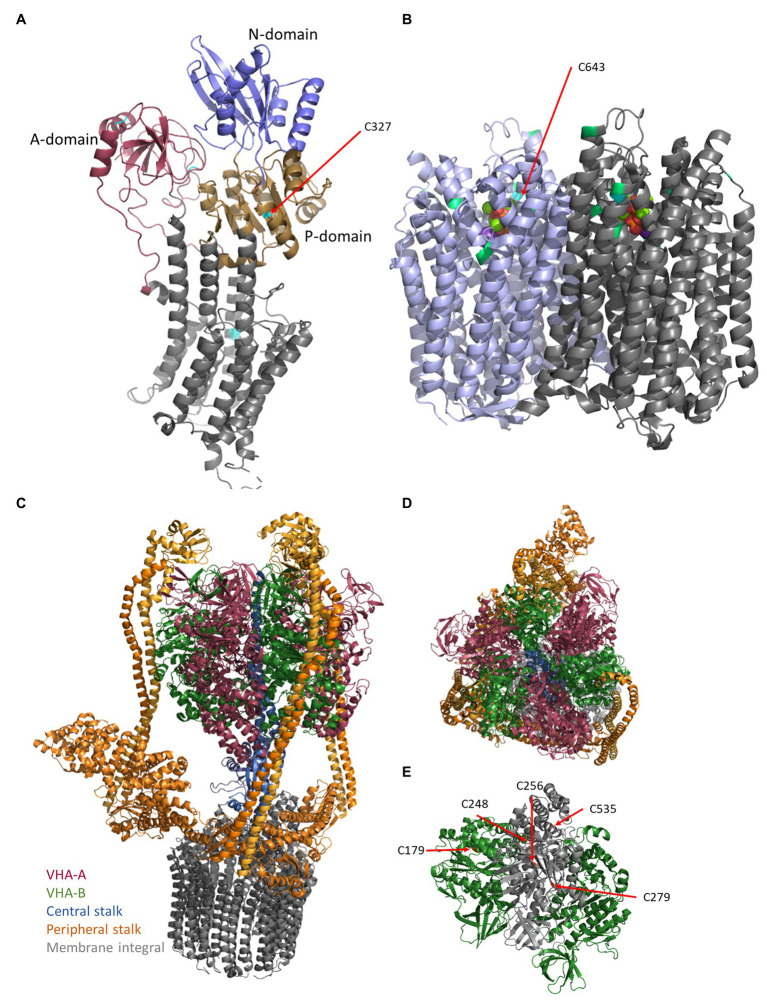
Structures of the proton pumps. The structures of plasma membrane-ATPase (PM-ATPase) AHA2 of *Arabidopsis thaliana* **(A)** and vacuolar pyrophosphatase (V-PPase) from *Vigna radiata* **(B)** base on the pdb-files 5KSD (Croll and Andersen, 2016; Focht et al., 2017) and 6AFS (Tsai et al., 2019), respectively. **(A)** The PM-ATPase consists of 10 transmembrane domains and a large cytoplasmic C-terminal domain (Almeida et al., 2017; Nguyen et al., 2020). In detail, the structure of the cytosolic domain can be divided into the actuator- (A-domain), the nucleotide-binding- (N-domain), which is embedded in the phosphorylation domain (P-domain), and a disordered C-terminal region. ATP binds to the nucleotide-binding domain, which in turn moves closer to the phosphorylation domain, thereby forming the catalytic site. Cysteine residues are indicated by cyan coloring. **(B)** The V-PPase (EC 3.6.1.1) functions as FIGURE 1homodimeric proton pump of 160 kDa at the tonoplast and acidifies vacuoles, in particular, vacuoles of expanding cells (Smart et al., 1998; Maeshima, 2001). It consists of 16 transmembrane domains, of which six helices are required for proton transport and cytosolic domains form five Mg^2+^-binding sites in *V. radiata* (Tsai et al., 2019). The 14-3-3 binding sites (green) and the conserved Cys643 (cyan) are highlighted. One molecule pyrophosphate is bound each monomer and visible as balls in the center. **(C)** The vacuolar-type ATPase (V-ATPase) consists of the membrane integral sector V_O_ (VHA-a, VHA-c, VHA-c”, VHA-d, and VHA-e) and the membrane associated sector V_1_ (VHA-A–VHA-H), which represent the proton translocator (gray) and ATPase, respectively **(C)**. Proton transport occurs by rotation of a proteolipid ring formed by multiple copies of VHA-c and a single copy of VHA-c”. VHA-a contributes half channels as proton inlet and outlet pipes. The protons have accessed to a conserved glutamate residue of the proteolipids, protonated the amino acid, and become deprotonated after one approximately one turn by a positive barrier charge of VHA-a, which alters the pKa of the glutamate so that the proton is released into the lumen. VHA-d serves as a bearing between the proteolipid ring and the central stalk of V_1_. This central stalk (blue) is formed by VHA-D and VHA-F and transduces the sequence of conformational alterations due to ATP-hydrolysis within three copies of VHA-A into rotation. **(C,D)** VHA-A (red) and VHA-B (green) form a hexameric head around (red/green) the central stalk (blue) and are anchored to the membrane by three rigid peripheral stalks (each formed by VHA-E and VHA-G heterodimer, orange), which are crosslinked by VHA-C (orange) and VHA-H (orange) and anchored to the membrane *via* a cytosolic domain of VHA-a, so that VHA-a is essential for proton transport and avoids co-rotation of the head structure. **(E)** Within VHA-A, Cys256, Cys279, and Cys535 are highly conserved among all eukaryotes, Cys248 is plant-specific and the distance between Cys Cys256 and Cys248 is approximately 11 Å and would allow for disulfide formation. VHA-B bears Cys179, which is target of redox modification. The pdb-file 3j9t was used as template (Zhao et al., 2015).

The vacuolar pyrophophatase functions as homodimeric proton pump (Figure 1) at the tonoplast and acidifies in particular vacuoles of expanding cells (Smart et al., 1998; Maeshima, 2001; Segami et al., 2010). It enables the usage of other resources than ATP and increases the cellular energy-use efficiency (Munns et al., 2020), since pyrophosphate is a byproduct of multiple processes, such as protein, starch, and cellulose synthesis (Taiz, 1992). Two types of V-PPases are known, which differ in potassium and calcium sensitivity (Maeshima, 2000). Calcium-inhibition occurs through formation of CaPP_i_ as inhibitor or direct binding of Ca^2+^ as inhibitory ligand (Baykov et al., 1993; Sivula et al., 1999).

AVP1 of *A. thaliana* belongs to the type I V-PPases, which locate to the vacuole and are nearly Ca^2+^-insensitive, but potassium-sensitive (Almeida et al., 2017). The type II V-PPase AVP2, which is Ca^2+^-sensitive and potassium insensitive, was found at the Golgi in *A. thaliana* (Mitsuda et al., 2001). V-PPases keep the cytosolic pyrophosphate level low and avoid inhibition of gluconeogenesis by high pyrophosphate concentrations (Martinoia, 2018), but hyper activity of V-PPases has negative effects on cellular viability (Graus et al., 2018). During germination, V-PPases are of even higher importance than soluble PPases (Martinoia, 2018). AVP1 further localizes to the plasma membrane in companion cells of sieve elements and was suggested to function as pyrophosphate synthase. This inverse function might maintain the pyrophosphate homeostasis in this cell type and supports phloem respiration (Pizzio et al., 2015).

The V-ATPase consists of the membrane integral sector V_O_ and the membrane-associated sector V_1_ (Figure 1), which represent the proton translocator and ATPase, respectively. The active enzyme was identified at the TGN/EE and the vacuole and both isoenzymes can be differentiated by the present isoform of VHA-a: In *A. thaliana*, the vacuolar pump bears either VHA-a2 or VHA-a3, while the TGN-located pump bears VHA-a1 (Dettmer et al., 2006). Other proteins might contribute to the active complex, since interaction with the glycolytic aldolase has been identified, which links the proton pumping to the cellular energy state (Konishi et al., 2004; Schnitzer et al., 2011). The V-ATPase energizes the transport of solutes into and out of the vacuole together with the V-PPase. Interestingly, lack of the vacuolar isoenzyme is tolerated by the plant cells, as shown by a double knock-out of the vacuolar-specific subunits VHA-a2 and VHA-a3, but knockout of VHA-A, which is essential for all V-ATPases, is embryolethal. It has been postulated that its presence at the TGN/EE is essential due to cell wall components synthesis and transport, while its absence at the tonoplast can be compensated by V-PPase and the TGN-specific isoenzyme (Krebs et al., 2010).

## Coordination of Transport

In plants, PM-ATPase, V-PPase, and V-ATPase regulate cellular pH homeostasis in combination with other transporters. Coordination can be achieved by ionic conditions in the cytosol, in particular calcium ions and nitrate, cytosolic pH, nucleotides, malate, kinases, and phosphatases, redox conditions, and membrane potential. Assuming lack of coordination of transport processes at tonoplast and plasma membrane, the cytosolic solute concentration would putatively increase by 150 mM/min in guard cells during stomata opening (Cubero-Font and de Angeli, 2020).

The uptake of nitrate or ammonia is accompanied by uptake of other ions and results in transient change of the membrane potential of the plasma membrane. Ammonia stimulates the activity of the plasma membrane ATPase (Yamashita et al., 1995). Since the nitrate assimilation is proton-consuming, it might level the proton influx caused by nitrate influx (Feng et al., 2020). Excess of nitrate inhibits V-ATPase activity, and the electrochemical gradient at the plasma membrane enables nitrate efflux in order to balance the cytosolic pH (Feng et al., 2020). Thus, proton pumping is directly affected by nitrate so that it might contribute to coordination of proton pumps. This can be mediated by the chloride channel family CLC, which is responsible for chloride and nitrate transport. In *A. thaliana*, the family consists of seven members (CLC-a–g) and locates to the vacuole and the TGN (von der Fecht-Bartenbach et al., 2007). Feng et al. (2020) postulate that V-ATPase and CLC-a co-operate to balance the cytosolic pH, electrical balance also depends on potassium transport. Fine tuning of cytosolic pH is achieved by Alkali cation/proton antiporters like Na^+^/H^+^-antiporter (NHX) and CHX in response to environmental changes, NHX1 and NHX2 might further be a major source of proton leakage (Sze and Chanroj, 2018).

Within the cytosolic environment, ROS play an important role in both posttranslational modification and, consequently, intracellular signaling. In response to biotic and abiotic stress, ROS and also RNS (reactive nitrogen species) accumulate (Hasanuzzaman et al., 2020). Besides mitochondria, chloroplasts, and peroxisomes, ROS formation also occurs in the apoplastic space by plasma membrane NADPH oxidases [NOXs; or respiratory burst oxidase homologs (RBOHs)], cell wall-associated peroxidases (POXs), and oxalate oxidases (Torres et al., 1998; Kawano, 2003; Mittler et al., 2004; Asada, 2006; Sagi and Fluhr, 2006; Qi et al., 2017). NOXs were shown to be activated by cytosolic acidification to inhibit growth under conditions of acid stress (Bissoli et al., 2020). As the most stable form of ROS, hydrogen peroxide (H_2_O_2_) is capable of migrating across biological membranes *via* aquaporins (Bienert et al., 2006, 2007). H_2_O_2_ can either cause oxidative damage to proteins and DNA or act as a signaling molecule, thereby regulating plant physiological processes (Mittler and Berkowitz, 2001). The sulfur containing amino acids methionine and cysteine are particularly addressed by H_2_O_2_. Redox-based PTMs determine protein activity, binding affinity, localization, protein-interaction, and conformation (Valderrama et al., 2007; Spoel and Loake, 2011; Jacques et al., 2015; Zhang et al., 2020).

*In vitro*, V-ATPase activity is inhibited upon treatment with H_2_O_2_, nitric oxide, N-ethylmalmeide, iodacetamide, and oxidized glutathionine and thioredoxin (Hager and Biber, 1984; Hager and Lanz, 1989; Dietz et al., 1998; Tavakoli et al., 2001; Seidel et al., 2012). It has recently been demonstrated in cucumber that the plant V-ATPase activity can be fine-tuned by H_2_S as a counterpart to H_2_O_2_. Plants treated with H_2_S-source NaHS were upregulated in V-ATPase activity, whereas lowered H_2_S content reduced V-ATPase activity. This might be due to H_2_S-mediated release of inhibitory disulfide bridges (Kabala et al., 2019). Similarly, PM-ATPase activity was reported to be enhanced by H_2_S in soy bean roots (Wang et al., 2019).

VHA-A was identified as redox-sensitive subunit (Wang et al., 2012; Waszczak et al., 2014). In VHA-A, Cys256, Cys279, and Cys535 are highly conserved among all eukaryotes (Seidel et al., 2012). Although, Feng and Forgac (1994) observed disulfide bridge formation, the ATP-binding is not affected by disulfide bridge formation within subunit A. Accordingly, the absence of Cys279 or Cys535 residues does not abolish V-ATPase sensitivity toward H_2_O_2_, GSSH, and GSNO, whereas C256S mutants are mainly insensitive. Taken together, in *A. thaliana*, Cys256 mediates sensitivity of V-ATPase ATP-hydrolysis toward redox changes, while proton pumping activity is Cys256-independently inhibited by H_2_O_2_. Because Cys256, Cys279, and Cys535 could not be demonstrated to be involved in disulfide bridge formation in VHA-A, the close distance of S-sulfenylated Cys248 to Cys256 needs to be taken into account, although Cys248 is only conserved among plants (Figure 1). Grüber et al. (2000) reported the V-ATPase subunit VHA-B of insect midgut undergoing structural alteration upon non-reducing conditions. In VHA-B, only one cysteine (Cys179) is conserved among eukaryotes. Intramolecular disulfide formation has also been reported in barley subunit E that occurs upon oxidative treatment of V-ATPase *in vivo* and was stated to be involved in redox-dependent V-ATPase activity changes (Tavakoli et al., 2001). In *A. thaliana*, subunit VHA-E Cys134 and Cys186 are conserved among plant species, but do not occur in other organisms, suggesting a plant specific function (Tavakoli et al., 2001).

**Figure 2:**
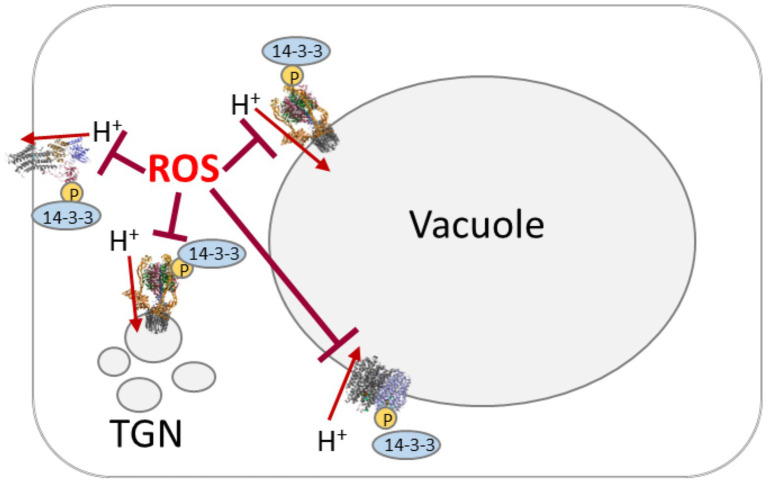
Scheme of proton pumping-coordination by reactive oxygen species (ROS) and phosphorylation/14-3-3 proteins. Proton pumps are target of oxidative inhibition by ROS. The activated state involves phosphorylation by cytosolic kinases (“P” in yellow circle) and subsequent binding of 14-3-3 proteins.

At the plasma membrane, NOX catalyzes electron transfer from NADPH to O_2_. Thereby, NOX generates apoplastic O_2_^ˉ^, but also acidifies the cytosol (Ramsey et al., 2009). In order to counteract cytosolic acidification and to provide H^+^ for apoplastic ROS scavenging processes, PM-ATPase has been proposed to be cooperatively regulated with NOX (Majumdar and Kar, 2018). Indeed, H_2_O_2_ concentrations up to 50 μM promoted PM-ATPase activity in *Vigna radiata*, whereas higher H_2_O_2_ concentration was inhibitory. Furthermore, in the presence ROS-scavengers and NOX inhibitors, PM-ATPase activity decreased, supporting the idea of H_2_O_2_-dependent activation of PM-ATPase (Majumdar and Kar, 2018). Involvement of H_2_O_2_ in upregulation of PM-ATPase has also been reported in *Populus euphratica* callus (Zhang et al., 2007) and in *Carex moorcroftii* callus (Li et al., 2011). However, plasma membrane H^+^-ATPase inactivation at higher H_2_O_2_ concentration might occur due to thiol oxidation (Lee et al., 2004; Beffagna et al., 2005; Majumdar and Kar, 2018). Cytosolic pH changes correlate with H_2_O_2_ accumulation; this data suggest PM-ATPase to adjust proton transport dependent on H_2_O_2_ accumulation (Beffagna et al., 2005). Five cysteine residues of AHA1 are conserved among plants (Figure 2), of which Cys327 is located in the cytosolic P-domain. Cys327 is conserved among all P2 and P3 ATPases in eukaryotes, shows a close distance to the phosphorylated aspartate residue and Mg^2+^-binding site, is redox-sensitive and thereby pointing to a redox regulatory role (Welle et al., 2021).

Redox control of V-PPase is poorly understood. AtAVP1 possesses seven cysteine residues that are conserved among plant species. First evidence of V-PPase being addressed by redox changes in *A. thaliana* came from (Zhen et al., 1994), who reported irreversible inhibition by N-ethylmaleimide (NEM) and 3-(N-maleimidylpropionyl) biocytin (MPB). Cys643 was shown to be exclusively affected by NEM, thereby inhibiting both PPi hydrolysis and H^+^ translocating activity (Figure 2). Nevertheless, substitution of Cys643 to alanine or serine has not changed total V-PPase activity, suggesting a rather regulatory or even the lack of an important function (Kim et al., 1995).

Proton pumps, potassium-channels, and CLC-proteins are target of phosphorylation. Kinases might act on proteins in different membranes, such as the receptor-like kinase KIN7, which locates to the tonoplast and the plasma membrane (Isner et al., 2018; van Kleeff et al., 2018; Subba et al., 2020) or the SOS2/SOS3 complex, which activates cation-proton-antiporters (NHX, CAX), V-ATPase, and PPase (Shabala et al., 2020). Other candidates are MAPKs, which might act on transporters, too (Cubero-Font and de Angeli, 2020). Together with membrane-associated phosphatases they seem to be key players of transport regulation: PP2A dephosphorylates CLC-a (Subba et al., 2020), PP2A-C5 interacts with the vacuolar CLCs -a, -b, -c, and –g (Hu et al., 2017). Inhibition of PP2C.D keeps PM-ATPase active (Spartz et al., 2017), what is in good agreement with auxin-dependent inactivation of PP2C-D1 manner by SMALL AUXIN UP RNA 19 (SAUR19) for regulating AHA1 and AHA2 in *A. thaliana* (Mangano et al., 2018; Ren et al., 2018). However, the subsequent binding of 14-3-3 proteins to phosphorylated proton pumps might be the real coordinating step (Figure 2). Indeed, Almeida et al. (2017) suggested that all three proton pumps are regulated by one mechanism with 14-3-3 proteins as a good candidate. 14-3-3 proteins act on all three proton pumps, mediating for instance blue light-activation of PM-ATPase and V-ATPase (Assmann et al., 1985; Klychnikov et al., 2007; Hsu et al., 2018; Nguyen et al., 2020). 14-3-3 proteins act as dimers and combining the 13 isoforms in *A. thaliana* allows for the formation of 91 homo- and heterodimers. Isoform-specifity has been analyzed for the V-PPases, where isoforms ν, μ, ο, and ι increased the activity and protected against inhibition by high levels of pyrophosphate (Hsu et al., 2018). Besides the high number of putative 14-3-3 dimers, a high number of eight putative binding sites were identified (Figure 1), even binding independent of phosphorylation was observed (Hsu et al., 2018). In case of the PM-ATPase, 14-3-3 proteins bind to the phosphorylated C-terminal domain in an activating manner, preventing the autoinhibitory function of the domain and enabling binding to the RIN4-complex, a negative regulator of plant immunity (Liu et al., 2009; Kaundal et al., 2017). This interaction is terminated by GCN4-mediated degradation of 14-3-3 proteins and RIN4-complex (Kaundal et al., 2017). Binding of 14-3-3 proteins can even result in hyperactive hexamers of PM-ATPase (Nguyen et al., 2020). 14-3-3 proteins also act on GORK, which is phosphorylated by CPK21 and then target of the 14-3-3 isoforms λ, χ, and ν (van Kleeff et al., 2018).

## Conclusion

Though more data are required on some regulatory mechanism, it is plausible that coordinated regulation of plant proton pumps occurs on several levels with respect to the growth conditions. Pathogen-defense likely involves ROS first as messenger and then as defense strategy and requires adjustment of the proton pumps according to the current state (Figure 2). Interestingly, non-protein thiols have been intensively analyzed for their effect on proton pumping, but the knowledge on the impact of protein thiols is still scarce.

Phosphorylation and 14-3-3 proteins mediate a day-night dependency of the proton pumping activity, adjusting for instance nitrate transport and assimilation to the diurnal requirements, besides the direct interaction between nitrate and proton pumps. The number of kinases, phosphatases, and 14-3-3 proteins result in a broad variety of combinations, pointing to high flexibility for adjustments of proton transport and cytosolic redox homeostasis. Finally, yet importantly, the contribution of anion and cation transport is essential for cytosolic pH-stat and maintenance of lumenal conditions and membrane potentials.

## Author Contributions

MC and TS have prepared the manuscript. MC focused on redox-regulation. TS focused on the general aspects and 14-3-3 proteins. All authors contributed to the article and approved the submitted version.

### Conflict of Interest

The authors declare that the research was conducted in the absence of any commercial or financial relationships that could be construed as a potential conflict of interest.

## Abbreviations

V-ATPase: vacuolar-type ATPase
PM-ATPase: plasma membrane-ATPase
V-PPase: vacuolar pyrophosphatase
NHX: Na^+^/H^+^-antiporter
